# The Future of Periodontal-Systemic Associations: Raising the Standards

**DOI:** 10.1007/s40496-017-0150-2

**Published:** 2017-07-12

**Authors:** P. Mark Bartold, Angelo Mariotti

**Affiliations:** 1 Department of Dentistry, University of Adelaide, Adelaide, SA 5005 Australia; 2 Department of Periodontology, The Ohio State University, 305 W. 12th Ave, Columbus, OH 43210 USA

**Keywords:** Periodontal medicine, Periodontal systemic inter-relationships, Disease Association Checklist, Bradford Hill criteria, STROBE Statement

## Abstract

**Purpose of Review:**

Periodontal medicine recognizes a “bidirectional” interaction between periodontitis and systemic conditions. Unfortunately, the facile ability to publish a periodontal-systemic association, regardless of biologic plausibility or rigorous scientific scrutiny, continues without abate.

**Recent Findings:**

The increasing number of periodontal-systemic associations corrupts the ability of dentists to distinguish which of the associations are spurious and which are valid.

**Summary:**

The use of a Disease Association Checklist creates a register for rational assessment of current disease associations. However, to diminish the publication of spurious periodontal-systemic observational associations, editors must demand that authors follow Bradford-Hill criteria and the STROBE Statement to ensure a stringent pathway to publication.

## Introduction

“Periodontal Medicine” was first proposed in 1996 as a broad term defining an emerging branch of periodontology that focussed on relationships between periodontal health/disease and systemic health/disease [1]. Now, 20 years later, the list of “associations” has burgeoned to unwieldy proportions, and it is time to consider criteria to evaluate the accuracy of the current reported associations and make recommendations concerning what future studies should be published.

**Fig. 1:**
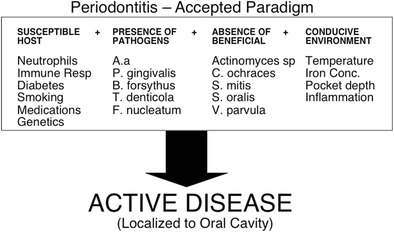
Conventional paradigm of periodontal disease whereby periodontitis arises in systemically susceptible individuals due to a chronic inflammatory reaction to subgingival plaque

**Fig. 2:**
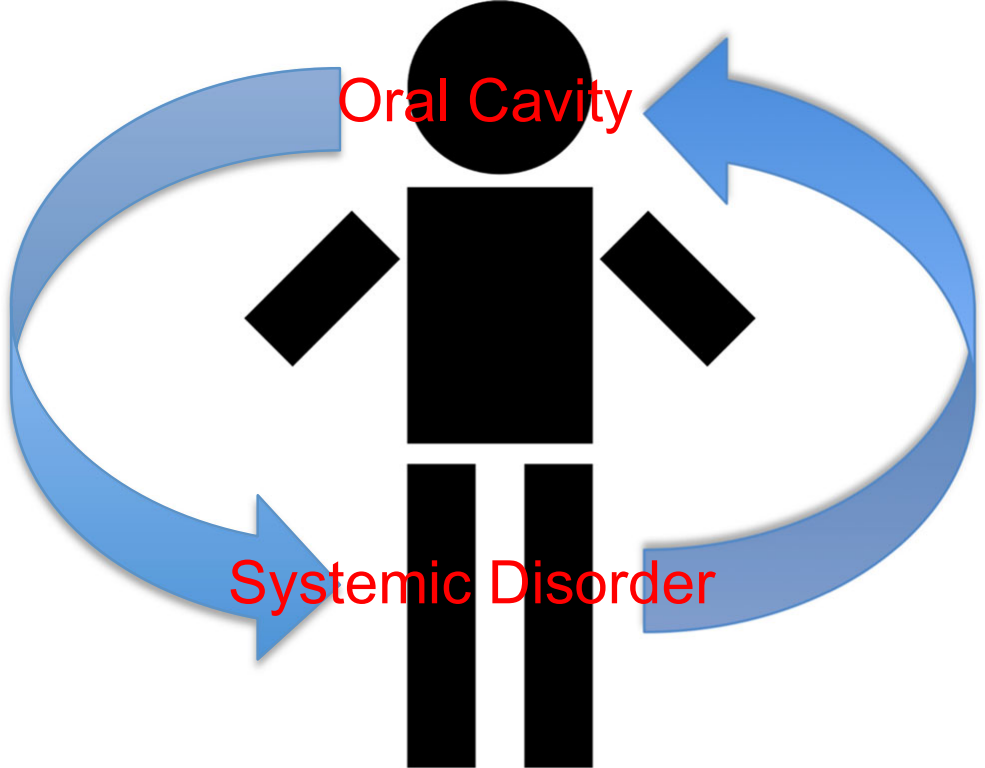
Inverted paradigm of periodontal disease whereby systemic conditions can influence periodontal status and conversely periodontal status can influence systemic conditions

Central to the tenet of periodontal medicine is the concept that periodontal inflammation and the periodontal microbiome contribute to the overall burden of inflammation at the systemic level that impacts the incidence, severity, and progression of many other chronic inflammatory conditions. As a result of this concept, our understanding of periodontal disease has been dichotomised. Moreover, one part consists of the conventional or accepted disease model in which periodontitis arises in systemically susceptible individuals due to a chronic inflammatory reaction to subgingival plaque (Fig. 1). The other or second paradigm has led to the conventional or diseased model being inverted [2] in which periodontitis has the potential to impact systemic conditions either through disease associations or disease causality (Fig. 2). Accordingly, periodontal medicine recognizes a “bidirectional” interaction between periodontitis and systemic conditions, whereby not only can periodontitis affect systemic health but also systemic health can affect periodontitis.

## Understanding Oral Health and Systemic Health Interrelationships

The ways in which periodontal health or disease may affect general health and well-being are largely unknown because of their complexity. There is emerging evidence to support a growing number of associations between periodontitis and chronic medical conditions. How valid are these associations considering the complexity of the biological process? Furthermore, how does this expanding universe of periodontal-systemic associations, independent of their legitimacy, become entrenched in the literature? To understand this, it is necessary to appreciate the method used by periodontal investigators to collect and publish evidence obtained from populations regarding relationships between periodontal disease and systemic disease.

## Observational Studies Are….Merely Observations

In the field of periodontal medicine, the use of observational studies has become the prime method to identify possible associations between periodontal diseases and systemic diseases. Therefore, understanding the strengths and limitations of observational studies is important in determining the veracity of periodontal-systemic associations.

An observational study interprets the effect of direct observations of subjects in their natural settings where the researcher has no control over variables. In other words, the assignment of subjects is not directed by the investigator but rather subject characteristics (e.g., age, sex, and disease status) are observed and recorded by the investigator. Observational studies allow access to situations and people to provide a real-world aspect to develop a hypothesis. With an observational study, the investigator can observe how individuals act together or separately and assess behavior or relationships based on selected metrics that are more reliable than self-reported outcomes. The frequency of outcomes in an observational study is usually measured or estimated as relative risks, rate ratios, hazard ratios, and odds ratios.

Although observational studies can identify associations and generate a hypothesis, they cannot demonstrate a cause and effect relationship. To confirm the validity of a hypothesis, experimental studies (e.g., randomized clinical trial) are necessary. Since observational studies are not considered experimental studies, they are subject to a variety of distinctive methodological problems. For example, many of the observed results are a consequence of individual preferences, practice patterns, and/or policy decisions [3]. Further, since individuals are not randomized into experimental and control groups, selection bias, information bias, investigator bias, confounding variables, effect modification, and chance can challenge the interpretation of the results from observational studies.

Despite the limitations of observational studies, these types of studies can offer compelling associations (e.g., smoking and lung disease) that can be confirmed by robust experimental data. However, relying on observational data alone to validate treatment policy is problematic. More specifically, observational studies have been suggested to often provide false research claims [4]. For example, observational studies supported use of coronary stents to reduce chest pain; however, stenting of coronary arteries for stable coronary artery disease was found to be no better than medical management of patients with this medical problem [5]. This is one example of numerous reversals of medical practice that were originally supported by observational studies [6]. The reasons for inaccuracies noted in observational studies usually occur when there are (1) small populations observed, (2) minor effect sizes, (3) a greater number and lesser preselection of tested relationships, (4) variations in designs, definitions, outcomes, and analytical modes, (5) financial and other interests and prejudice, and/or (6) scientific teams chasing a statistical significance in what is a hotly contested research area [4].

## The Expanding Data Set: Helpful or Ineffectual?

The premise of a healthy (periodontal) mouth being essential for general well being is the fundamental basis of periodontal medicine and is a reasonable working model to provide high quality care for patients. This concept should drive future clinical and scientific endeavors in the years to come and must be based on sound biologic plausibility and not trivial whim. Therefore, extreme care must be taken not to over interpret, or misrepresent, the evidence gained from a single, observational study. It should go without saying that for any new developments, such as the relationships between periodontal diseases and systemic conditions, considerable care should be exercised to ensure studies are well focussed and based on sound principles with biologic rationale. Unfortunately, it appears that this may not always be the case, and concern should be expressed over the increasing number of studies that appear to be poorly conceived with little or no thought related to the biologic plausibility of the relationships being studied. The expanding list of periodontal-systemic associations should be of concern as more and more trivial associations seem to be reported with little regard for biologic plausibility or rigorous scientific scrutiny. Indeed, concerns over the publication of spurious associations in oral epidemiological research have been present for over a decade [7•].

Currently, there are over 100 reported periodontal-systemic associations, and all of these relationships have been identified by observational studies. At this time, only one association (periodontal disease and adverse pregnancy outcomes) has been investigated using experimental studies with distinct, objective, clinical endpoints (not surrogate values), and the experimental studies failed to confirm a relationship [8]. Another interesting conundrum is that all periodontal-systemic associations appear to work through similar mechanisms that involve periodontal (a) regulatory substances (e.g., cytokines) and/or (b) organisms (e.g., *Porphyromonas gingivalis*) that are hypothetically transported in tissue fluids (e.g., blood) to stimulate specific cells or tissues distant from the periodontal apparatus (e.g., cardiac tissue). This ubiquitous mechanism raises the question concerning whether it is possible to have over 100 different periodontal-systemic interactions all operating by the same process. What can you believe?

## Are Periodontal-Systemic Associations Risk Assessments, Causations, Syndromes, Syndemic Epidemics, or Spurious?

In the context of periodontal and systemic health associations, if periodontal disease is believed to cause a disease, then it must always precede the occurrence of the disease. Clearly this is often not the observed case for periodontal diseases because not all people who have a periodontal disease suffer from any of the known, and proposed, systemic conditions all of the time. However, it is very likely that subsets of patients with periodontal disease could represent a group of individuals at increased risk for manifesting a variety of inflammatory-associated diseases. For example, chronic periodontitis may be a risk predictor (a characteristic associated with elevated risk but not necessarily an element in the causal chain of events) for coronary artery disease. In this context, disease associations may be just as important as causality relationships in the sense that identifying individuals at risk of having one or more systemic conditions may help in their overall clinical management.

In the same way that multiple diseases may align with each other, the concept of syndromic relationships arises. Given that a syndrome is a group of symptoms that collectively indicate or characterize a disease, psychological disorder, or other abnormal condition, various periodontal and systemic conditions could fall into the groupings associated with a syndrome [9].

In a similar manner, it has been proposed that associations of periodontal diseases and systemic diseases may be a syndemic relationship [10]. Syndemic refers to a set of two or more linked health problems that interact synergistically to cause an excess burden of disease in the population. Within this context, it would seem reasonable to believe that some associations between periodontal disease and systemic diseases may be of a syndemic nature.

In an attempt to clarify the current evidence for relationships between periodontitis and systemic diseases, a joint summit of the European Federation of Periodontology and the American Academy of Periodontology met in 2012. [11•]. Specifically, relationships between periodontitis and cardiovascular disease, diabetes mellitus, adverse pregnancy outcomes, respiratory disease, chronic kidney disease, rheumatoid arthritis, cognitive impairment, obesity, metabolic syndrome, cancer, and other associations were considered. From the deliberations of this meeting, it was concluded that there was evidence in the literature to support the concept that periodontitis is associated with some, but not all, of the diseases and conditions reviewed.

In the current climate of periodontal-systemic relationships, where numerous associations are being reported, the association of periodontal and systemic diseases should be viewed in the context of (a) the risk markers that may bind them together and (b) experimental (not just observational) data that define the relationship between them. Furthermore, dentists can begin to ascertain how periodontal diseases relate to systemic disorders using a “Disease Association Checklist” (Table 1) to create a simplified register of criteria that allows for rational assessment of disease associations. When all of the available data are assembled and imported into such a sieve, patterns will emerge with regard to how robust the evidence for various periodontal and systemic interrelationships can be accepted to support meaningful relationships. By using the Disease Association Checklist, supportive evidence exists for an association between periodontitis and diabetes, cardiovascular disease and rheumatoid arthritis, equivocal evidence of an association between periodontal disease and obesity, or adverse pregnancy outcomes and negligible evidence for osteoporosis (Table 2).

**Table 1:** How periodontal diseases relate to systemic disorders using a “Disease Association Checklist”

Disease Association Checklist
Biologic plausibility
Strength of association
Effect of periodontal treatment on systemic condition
Effect of treatment of systemic condition on periodontitis

**Table 2:** Various periodontal and systemic interrelationships

	Diabetes	Obesity	Adverse pregnancy outcomes	Cardiovascular disease	Osteoporosis	Rheumatoid arthritis
Biological plausibility	Yes	Yes	Questionable	Yes	Yes	Yes
Strength of association	Yes	Emerging	Poor	Yes	No	Yes
Effect of periodontal Treatment on disease Condition	Yes	Nil	None	Equivocal/none	No	Emerging
Effect of treatment of disease condition on periodontitis	Yes	No	No	No	No	Emerging

## Periodontal Medicine Epistemology

In 2000, the US Surgeon General stated that “Oral health and general health should not be interpreted as separate entities” and furthermore that “oral health is a critical component of health and must be included in the provision of health care and the design of community programs” [12••]*.* Although this perception forms the fundamental tenet of periodontal medicine, research in this area would benefit from an epistemological approach whereby the nature of our knowledge, as well as the extent of our knowledge, would be fully scrutinized and not trivialized. To achieve this goal, dentists must be confident that reported periodontal-systemic associations are convincing prior to any experimental studies being executed to ascertain their authenticity.

As mentioned earlier, observational studies can be interesting but isolated, they are of limited value since studies such as these are not sufficiently reliable to inform patient care or health policy. It should go without saying that any association between periodontal diseases and systemic conditions identified through observational studies must follow a more stringent pathway to publication. To this end, the Bradford Hill criteria [13••] and the STROBE (STrengthening the Reporting of Observational Studies in Epidemiology) statement [14••] must be employed for publication of any periodontal-systemic observational study. The Bradford Hill criteria form evidence guidelines to establish a causal relationship of putative observations and effects. Bradford Hill criteria require biological plausibility, data consistency, specificity and coherence, proper temporal relationships, biological gradients, experimental reversibility, and other similar biological precedents. The STROBE Statement affects the conduct and dissemination of observational studies. STROBE Statements require improved quality of study reporting, data sharing, transparency concerning what was planned and what was done, protocol publication, and registration of the study. Both Bradford Hill and STROBE substantially improve the quality of the observational data and should eliminate observational studies that are poorly conceived, have no biological plausibility, exhibit bias, and have other significant study limitations. Therefore, to ensure the quality of the observational study for publication, both Bradford Hill criteria and the STROBE Statement must be required by editors for publication of observational investigations claiming links between periodontal diseases and systemic diseases.

## Conclusions

The development in periodontics of periodontal medicine as a sub-discipline has resulted in an inversion of our current thinking in periodontology. Traditionally, periodontal treatment has focused on preserving or restoring the structure, function, and esthetics of the dentition; however, with the emergence of how periodontal inflammation and infection may impact overall health and well being, focus is turning towards preventing untoward effects of periodontal disease on overall health. As the possible relationships between oral and systemic health gain coverage in the public, we must not raise false hopes of treatment resulting from inappropriate studies containing irrelevant or trivial associations. In the current publication environment, a Disease Association Checklist will be beneficial in the rational assessment of various associations. However, considerable effort must be expended to ensure only robust and genuine associations are published and later pursued by experimental studies. By applying the Bradford Hill criteria and the STROBE Statement, we propose that only highly probable relationships be published and further studied. To date, only a few conditions show a possible interconnectivity with periodontal disease. In this context, all of the periodontal-systemic associations must be carefully evaluated, and those that cannot meet the standards should not be credibly considered as a plausible and therefore realistic relationships. Clearly, there is a need to “raise the bar” for any published periodontal-systemic observational study.
